# Prenatal Dichlorodiphenyldichloroethylene (DDE) and Asthma in Children

**DOI:** 10.1289/ehp.8127

**Published:** 2005-07-18

**Authors:** Jordi Sunyer, Maties Torrent, Laura Muñoz-Ortiz, Núria Ribas-Fitó, Daniel Carrizo, Joan Grimalt, Josep M. Antó, Paul Cullinan

**Affiliations:** 1 Unitat Recerca Respiratòria i Ambiental, Institut Municipal d’Investigació Mèdica, Barcelona, Spain; 2 Universitat Pompeu Fabra, Barcelona, Spain; 3 Area de Salud de Menorca, IB-SALUT, Menorca, Spain; 4 Environmental Chemistry, Consejo Superior de Investigaciones Científicas, Barcelona, Spain; 5 Department of Occupational and Environmental Medicine, Imperial College, London, United Kingdom

**Keywords:** asthma, atopy, children, DDE dichlorodiphenyldichloroethylene, organochlorines

## Abstract

Prevalence of asthma increases with increasing dichlorodiphenyldichloroethylene (DDE) levels. However, the effect of early-life exposure, the fundamental window of exposure, is unknown. We assessed the association between prenatal DDE and other organochlorine compounds, and atopy and asthma during infancy. All women presenting for antenatal care in Menorca (Spain) over 12 months starting in mid-1997 were invited to take part in a longitudinal study; 482 children were subsequently enrolled, and 468 (97.1%) provided complete outcome data up to the fourth year of study. Prenatal exposure of organochlorine compounds was measured in cord serum in 405 (83%) children. Asthma was defined on the basis of wheezing at 4 years of age, persistent wheezing, or doctor-diagnosed asthma. We measured specific immunoglobulin-E (IgE) against house dust mite, cat, and grass in sera extracted at 4 years of age. DDE (median = 1.03 ng/mL) was detected in all children, as well as hexachlorobenzene (0.68 ng/mL) and polychlorobiphenyls (0.69 ng/mL). Wheezing at 4 years of age increased with DDE concentration, particularly at the highest quartile [9% in the lowest quartile (< 0.57 ng/mL) vs. 19% in the highest quartile (1.90 ng/mL); relative risk = 2.63 (95% confidence interval 1.19–4.69), adjusting for maternal asthma, breast-feeding, education, social class, or other organochlorines]. The association was not modified by IgE sensitization and occurred with the same strength among nonatopic subjects and among those with persistent wheezing or diagnosed asthma. DDE was not associated with atopy alone. Prenatal exposure to DDE residues may contribute to development of asthma.

Dichlorodiphenyltrichloroethane (DDT) was extensively used around the world as an insecticide from the 1940s until the end of the 1980s. Today, it is still widely sprayed in developing countries for disease-vector control (Wendo 2004). DDT is rapidly metabolized to 1,1-dichloro-2,2-bis(*p*-chlorophenyl) ethylene [*p*,*p*′-DDE (dichlorodiphenyl-dichloroethylene), hereafter DDE], which is a persistent, highly lipophilic chemical that can be detected throughout the world in sediments and in the food chain. Humans are exposed mainly through foods, and infants through the placenta and breast-feeding.

Decreased lymphocyte responses were associated with DDE in wildlife species (Lahvis et al. 1985) and in experiments with rats and mice (Banarjee 1987a, 1987b; Rehana and Rao 1992). In humans, DDE was associated with changes in cellular and humoral immunity (Cooper et al. 2004; Vine et al. 2001) and particularly with changes in T-cell–mediated immune cytokines related with allergy, such as interleukin-4 (Bilrha et al. 2003; Daniel et al. 2002). Similar effects have been found with other organochlorine compounds, such as hexachlorobenzene (HCB) (Michielsen et al. 1999) and polychlorinated biphenyls (PCBs) (Van Den Heuvel et al. 2002). Japanese children with Yusho disease, from exposure to high levels of PCBs, showed a high frequency of respiratory symptoms (Nakanishi et al. 1985). In a cross-sectional study among school children in Germany, DDE was strongly related with increases in total immunoglobulin E (IgE) and asthma (Karmaus et al. 2001, 2003). An increase of asthma mortality and asthma prevalence in adults was found among an older cohort of DDT sprayers (Beard et al. 2003), and the prevalence of wheeze increased with a variety of pesticides among current applicators (Hoppin et al. 2002). These studies, however, were unable to measure the prenatal exposure that is probably the fundamental window of exposure related to subsequent health events (Gluckman and Hanson 2004).

Menorca is one of the Balearic Islands in the northwest Mediterranean Sea, which has no local pollution sources. Here a general population birth cohort was set up in 1997 within the Asthma Multicenter Infants Cohort study (Polk et al. 2004). Our aim in this study was to assess the association of cord serum levels of DDE and other organochlorine compounds with atopy and asthma during early childhood.

## Materials and Methods

All women presenting for antenatal care in Menorca over 12 months (starting in mid-1997) were recruited; 482 children were subsequently enrolled, and 468 (97.1%) provided complete outcome data up to the fourth-year visit; of these children, 405 (84%) had organochlorine compounds in cord serum measured. Blood was drawn at 4 years of age in 360 children, 306 of whom had IgEs and peripheral white blood cells measured. Asthma was defined based on wheezing at 4 years of age, persistent wheezing, or doctor-diagnosed asthma. The outcome of interest was the presence of wheeze at 4 years of age or absence each year to this age. Wheezing was described on each interviewer-led annual questionnaire as “whistling or wheezing from the chest, but not noisy breathing from the nose.” One or more episodes of wheezing over 12 months constituted wheezing during any given year. Forty-seven children had wheeze at 4 years of age, 42 of whom (89.4%) did so also in a preceding year [persistent wheeze (Martinez et al. 1995)]. Parental report of doctor-diagnosed asthma at 4 years of age was alternatively used as outcome. Specific IgE against house dust mite (*Der p1*), cat (*Fel d1*), and grass was measured using the CAP method, with levels > 0.34 kU/L being considered positive. We defined atopy as a positive value to any of the allergens. The study was approved by the corresponding ethical committees, and written informed consent was obtained from the parents of all children.

Prenatal DDE and other organochlorines were measured in cord serum by gas chromatography (GC) with electron capture detection and GC coupled to chemical ionization negative-ion mass spectrometry (Sala et al. 2001).

Parents were invited to undergo skin prick testing to determine their atopic status. A wheal of ≥ 3 mm (mean of perpendicular measures) to any allergen in the presence of a positive histamine control and a negative un-coated control constituted a positive skin test. A positive skin test to at least one allergen was considered indicative of atopy (*Der p1*, *Fel d1*, or grass pollen).

The following variables came from a questionnaire administered to the pregnant mothers: number of asthmatic parents, maternal smoking, parity, education, and social class. The U.K. Registrar General’s 1990 classification was used to classify social class according to mother’s employment (Liberatos 1988). Antibiotic use, lower respiratory tract infection (LRTI), and breast-feeding data came from the first-year questionnaire. LRTI was defined as a positive response to the question “Has a doctor ever said that your [child] has had a chest infection?” Mothers reported type and duration of breast-feeding. Fish consumption was excerpted from the food frequency questionnaire filled in during pregnancy. The children’s birth weight and sex were obtained from information collected at birth.

We measured the association between DDE and wheezing by relative risk (RR) estimated using binomial regression. The RR was adjusted for known risk factors of childhood asthma (Polk et al. 2004) in a multivariate model. DDE was log-transformed to normalize its distribution; it was also categorized by quartiles of its distribution. Linear dose–response relationships were assessed using general additive modeling and tested with DDE as a continuous variable (vs. discrete variable) in the regression model. We performed stratification by atopy to specify the type of asthma. Analyses were carried out with Stata version 8 (StataCorp, College Station, TX, USA).

## Results

Wheezing at 4 years of age was reported for 11.6% of all children, and absence of wheezing at any age in 41.8% of all children. Specific IgE to common allergens was positive in 12.6% of children who gave blood at 4 years of age (11.7% to house dust mite, 1.0% to cat, and 2.0% to grass pollen). The average white blood cell count was 8,453 cells/mL (range, 3,900–16,900 cells/mL), and the geometric mean of eosinophil percent was 3.04% (0.2–21.2%). The geometric mean of eosinophil counts was 244 cells/mL (25–2,099 cells/mL). Levels of organochlorines at birth are shown in Table 1. All children were born with quantifiable levels of DDE and PCB compounds.

DDE concentration increased significantly with maternal age (Table 2). Parity, high education, high social class, and low birth weight increased moderately with increasing levels of DDE, although in a non-statistically significant way. Fish consumption during gestation was poorly related with DDE. There were geographic differences in DDE within the island not explained by residence in a rural area or on a farm or by social class, education, or fish consumption. Maternal asthma, smoking, male sex, low gestational age, and no breast-feeding showed an association with wheezing at 4 years of age but not with DDE levels.

Wheezing at 4 years of age increased with DDE concentration, particularly at the highest quartile, which was also found for persistent wheezing (Table 3). Specific IgE was not associated with DDE, which also occurred after selecting a more stringent cutoff of atopy (CAP > 0.70) (*p* = 0.11). Eosinophil counts were higher in the last quartile of DDE, but the association was not significant. Stratification of the association between wheezing at 4 years of age and DDE by atopy did not suggest an effect modification.

DDE maintains an association with wheezing after adjusting for potential confounders [both as continuous variable (*p* = 0.002) and in the upper quartile (*p* = 0.01) (Table 4)]. Inclusion of location, residence on a farm, and maternal age did not change the association with DDE, nor did exclusion of any of the other variables in the model or the alternative use of maternal social class instead of education. This association was invariant among children sensitized to IgE or nonsensitized (*p*-value for interaction = 0.50). Table 4 does not show the adjusted association of wheezing with DDE among the IgE-sensitized children, given their small number (*n* = 37). Nevertheless, the fact that the unadjusted association between wheezing and DDE among the sensitized children [RR per each doubling of the concentration = 1.30; 95% confidence interval (CI), 0.91–1.86] was of a magnitude similar to that of the association for all the subjects reinforces a lack of interaction between DDE and atopy. All these findings were also observed for persistent wheeze. The use of doctor-diagnosed asthma (occurring in 1.9% of children) instead of wheezing as the outcome variable also resulted in a positive association (RR = 1.46; 95% CI, 0.92–2.32 ), although it was nonsignificant (*p* = 0.10). The association between wheezing and DDE was not modified by fish consumption, maternal asthma, or length of breast-feeding. The negative association between wheezing at 4 years of age and duration of breast-feeding was not modified by levels of DDE at birth (data not shown).

HCB (RR = 0.96; 95% CI, 0.69–1.30) per each doubling of the concentration, and PCBs (RR = 0.99; 95% CI, 0.81–1.21) did not show a significant association with wheezing (nor in quartiles), and their inclusion in the model with DDE did not change the association of DDE with wheezing.

## Discussion

Wheezing at 4 years of age increased with increasing levels of DDE at birth. This association occurred independently of specific IgE. An association between DDE and asthma at school age has already been reported by Karmaus et al. (2001) in a German population with a lower DDE burden (median of 0.30 ng/mL) than in the present study (1.03 ng/mL). Karmaus et al., however, measured both DDE and asthma at the same time, procedures that preclude measurement of prenatal exposure, which is probably the fundamental window of exposure related with the further health events (Gluckman and Hanson 2004).

Two pathways—immunologic and/or hormonal—could be involved in the relationship between DDE and asthma. The immunologic effects of DDE exposure have been suggested by many studies, although its mechanisms remain unclear. Several could be implicated. In humans, DDE was associated with changes in immune cells (Vine et al. 2001), immunoglobulins (Cooper et al. 2004; Vine et al. 2001), and cytokines (Bilrha et al. 2003; Daniel et al. 2002). DDE interferes with hormonal receptors and mimics estrogen activity (Rogan and Ragan 2003), which might modulate immunologic responses (Salem et al. 2000). Nevertheless, sexual hormones have been related to asthma by routes other than immunomodulation, such as in postmenopausal asthma, by unknown mechanisms (Barr et al. 2004).

In the children of our study, we did not find any association with peripheral total cell counts or with subtypes (data not shown). Only the number of peripheral eosinophils increased among the children in the highest quartile of DDE, although the difference was not statistically significant. Eosinophils participate in the underlying inflammatory responses of asthma (Bousquet et al. 1990). Yusho children exposed to PCBs who had respiratory diseases showed an increase of Clara cells in bronchioles (Nakanishi et al. 1985), which we did not investigate.

We did not find an association between DDE and specific IgE, in contrast to a study in school children measuring total IgE (Karmaus et al. 2001, 2003). A lack of association with IgE in our study could be due to the young age of our children, because expression of IgE sensitization to common aeroallergens increases with age during childhood (Jackola et al. 2003). An alternative explanation could be that the association between DDE and asthma does not involve the immunologic cells related to specific IgE production. The unmodified association between DDE and wheezing found among nonatopic children strengthens this possibility. Two studies on other organochlorines, such as PCBs and dioxins, found a negative association with allergic reactions in children (Weisglas-Kuperus et al. 2000) and IgE sensitization in rats (Luebke et al. 2001). A final explanation could be a discordant association between total and specific IgE. In neonates, organochlorines increased cord total IgE (Reichrtova et al. 1999).

A potential decreased response to viruses and bacteria due to DDE has been assessed in epidemiologic studies in children, but with some inconsistent results. Among 199 Inuit children highly exposed to organochlorines, a moderate increase of acute infections during the first year of life was reported (Dallaire et al. 2004), but not in 343 German school children (Karmaus et al. 2003) nor in 207 Dutch infants (Weisglas-Kuperus et al. 1995). We did not find any effect of DDE on wheezing occurring only before 3 years of age (data not shown), a probable marker of LRTIs. Coincidentally, LRTI during the first year measured by questionnaire was not associated with DDE (50% of the children in the lowest quartile of DDE had LRTI vs. 48% in the upper quartile).

DDE is an agricultural and industrial residue basically incorporated through diet. Fish are an important source of persistent organic compounds, but at the same time are also the basis of fundamental oligoelements. Parents of the children in the present study had a high intake of fish (more than half of the mothers had fish more than two times per week during pregnancy). However, fish intake has not been related to DDE levels, perhaps because of a small variability in our population (only 9% of our mothers ate fish less than once per week during pregnancy). We did not find any relationship between fish intake and wheezing at any of the DDE concentrations. Virtually all categories of foods might be contaminated by DDE, particularly fish and vegetables (Shafer and Kegley 2002). However, the food products primarily related to DDE have a protective role in asthma (Farchi et al. 2003), and therefore the present findings are unlikely to be explained by a residual confounding by maternal food patterns during gestation. Margarine and butter, previously related to wheezing (Farchi et al. 2003), were not associated with cord DDE in Menorca (*p* > 0.6). Food patterns did not explain the geographic differences in DDE levels in children.

Breast-feeding is an important way of ingesting organochlorines during infancy. At the same time, breast-feeding is negatively associated with wheezing at 4 years of age (Oddy and Peat 2003). The stratification of breast-feeding duration by prenatal levels of DDE did not modify the association between breast-feeding and wheezing, suggesting that the postnatal effects of DDE (incorporated through breast-feeding) are probably less relevant than prenatal exposure, as some authors have suggested for neurodevelopment (Nakai and Satoh 2002).

The risk factors other than DDE associated with wheeze in the present study are those already known to play a role in asthma inception (Polk et al. 2004).

A potential limitation of the present study is nonresponse (17%). However, in most cases subjects were not included because of the small quantity of sera in the repository aliquots of cord serum. The quantity of blood was unlikely to be related to DDE levels, and participants had the same rate of wheezing as did nonparticipants (*p* = 0.54). Thus, non-response is unlikely to have introduced bias. The proportion of subjects lost in the analysis of atopy was larger, because around 25% of children did not provide blood at 4 years of age. However, provision of blood was unrelated to DDE concentration (*p* = 0.89). Selection of children could not explain the differences in DDE levels by area of residence given the lack of a geographic pattern in the nonrespondents. The geography of DDE in Menorca is unknown, but the uniformity and small dimensions of the island suggest that it is unlikely that environmental exposures play a role. Nevertheless, a further environmental study might be of interest.

Overall, the present results suggest that prenatal exposure to DDE, the organochlorine residue with the highest levels in newborns from Menorca, may contribute to the incidence of asthma. With regard to DDE, Menorca may be considered representative of areas with low background pollution because there are no local sources of DDT release. These results should be considered when evaluating the risk benefits of spraying DDT in antimalarial campaigns, because the debate about its current use in developing countries with endemic malaria remains open (Chen and Rogan 2003; Wendo 2004).

## Figures and Tables

**Table 1:** Distribution of DDE (ng/mL) and other organochlorine values in cord serum by percentiles (*n* = 405).

	Minimum	25th	50th	75th	Maximum	Geometric mean
*p*,*p*′-DDE	0.04	0.57	1.03	1.94	19.54	1.06
HCB^a^	0.14	0.46	0.68	1.02	9.82	0.70
PCBs^b^	0.007	0.50	0.69	0.98	12.07	0.66

a Percent not detected = 1.2 %.

b Sum of congeners PCB-28, PCB-52, PCB-101, PCB-118, PCB-153, PCB-138, and PCB-180.

**Table 2:** Distribution (in percentage or mean) of women and children in the different quartiles of DDE concentration in cord serum with regard to the selected variables.

	*p*,*p*′-DDE (ng/mL)			
Characteristic	< 0.57 (*n* = 102)	0.57–1.03 (*n* = 100)	1.03–1.90 (*n* = 101)	> 1.90 (*n* = 102)
Mother (%)				
Age [mean (years)]*	27	28	29	30
Maternal asthma	9	4	7	9
Maternal atopy	45	32	31	40
Smoking during pregnancy	22	26	22	22
Parity (first)	52	50	47	46
Education				
Less than primary	9	6	4	8
Primary	60	47	48	54
University	8	13	17	17
Social class				
Professional/managerial	9	13	16	13
Manual partly skilled	21	14	11	17
Unemployment and housewife	27	17	21	15
Fish consumption during pregnancy				
< 1 per week	15	8	7	8
≥ 2 per week	52	60	55	49
Location (east)*	56	49	51	33
Rural area	15	9	13	11
Living on a farm	7	6	8	7
Child (%)				
Sex (male)	59	51	38	56
Gestational age [mean (weeks)]	40	40	39	40
< 37 weeks	5	3	3	5
Birth weight [mean (g)]	3,255	3,235	3,130	3,160
< 2,500 g	5	3	7	5
Breast-feeding	76	88	86	79
> 20 weeks	42	59	50	40
Weeks of exclusive breast-feeding (mean)	12	22	20	15

* *p*-Value for trend < 0.05.

**Table 3:** Distribution of wheezing, atopy (specific IgE > 0.34 kU/L), and eosinophil counts at 4 years of age according to quartiles of DDE in cord serum.

	*p*,*p*′-DDE (ng/mL)					
	< 0.57	0.57–1.03	1.03–1.90	> 1.90	RR (95% CI)^a^	*p*-Value^a^
Wheezing						
Never	56.1	54.0	53.5	38.5	1	
Persistent^b^	6.8	8.0	10.9	15.7	1.26 (1.04–1.54)	0.01
At 4 years of age	8.8	8.0	10.9	18.6	1.31 (1.09–1.58)	0.007
Atopy	16.7	13.7	9.5	10.7	0.92 (0.73–1.17)	0.51
Eosinophils (cells/mL)^c^	237	250	218	274	1.09 (0.96–1.25)	0.20
Wheezing at 4 years of age by atopy						
Atopic	33.3	0.0	28.6	62.5	1.30 (0.91–1.86)	0.14
Nonatopic	6.8	6.3	10.4	16.4	1.37 (1.06–1.79)	0.02

Values for *p,p*′-DDE presented as percentage except eosinophil counts/mL.

a Unadjusted RR, 95% CI, and *p*-value per each doubling of DDE.

b Wheezing at 4 years of age and in a previous year.

c Geometric mean, RR on having eosinophil > 340 cells/mL, which corresponds to a percentage of total cells > 4% and which occurred in 34% of children.

**Table 4:** Adjusted RR (95% CI) between DDE in cord serum and wheezing at 4 years of age.

Characteristic	All	Nonatopic
*p*,*p*′-DDE^a^	1.32 (1.13–1.54)	1.30 (1.05.1.62)
Maternal asthma	2.62 (1.46–4.71)	3.45 (1.18–10.10)
Maternal smoking	1.48 (0.89–2.47)	1.03 (0.51–2.10)
Parity (≥ second child)	1.18 (0.69–2.02)	1.54 (0.74–3.24)
Maternal education		
Primary	0.62 (0.26–1.46)	0.36 (0.13–1.04)
Secondary	0.80 (0.32–1.98)	0.37 (0.11–1.19)
High	0.29 (0.08–1.12)	0.17 (0.03–0.89)
Male	2.03 (1.15–3.57)	2.84 (1.21–6.68)
Gestational age (weeks)	0.87 (0.81–0.95)	0.90 (0.82–1.00)
Breast-feeding	0.57 (0.33–0.99)	0.34 (0.17–0.69)
*p*,*p*′-DDE in quartile (ng/mL)^b^		
< 0.57	1	1
0.57–1.03	1.00 (0.41–2.43)	1.32 (0.37–4.70)
1.03–1.90	1.62 (0.70–3.74)	2.63 (0.96–7.20)
> 1.90	2.36 (1.19–4.69)	2.49 (1.00–6.19)

a Per each doubling of concentration.

b Adjusted for the variables in the table, except *p*,*p*′-DDE.
